# Exendin-4 alleviates β-Amyloid peptide toxicity *via* DAF-16 in a *Caenorhabditis elegans* model of Alzheimer's disease

**DOI:** 10.3389/fnagi.2022.955113

**Published:** 2022-08-05

**Authors:** Xiangwei Song, Yingqi Sun, Zhun Wang, Yingying Su, Yangkun Wang, Xueli Wang

**Affiliations:** ^1^School of Life Sciences, Changchun Normal University, Changchun, China; ^2^Plant Inspection and Quarantine Laboratory, Changchun Customs Technical Center, Changchun, China; ^3^School of Grain, Jilin Business and Technology College, Changchun, China

**Keywords:** Alzheimer's disease, Exendin-4, *Caenorhabditis elegans*, DAF-16, Amyloid-β peptide, neuroprotection

## Abstract

Epidemiological analyses indicate that type 2 diabetes mellitus (T2DM) is a risk factor for Alzheimer's disease (AD). They share common pathophysiological mechanisms. Thus, it has been increasingly suggested that several anti-T2DM drugs may have therapeutic potential in AD. Exendin-4, as a glucagon-like peptide-1 (GLP-1) receptor agonist, is an approved drug used to treat T2DM. In this research, the neuroprotective effect of Exendin-4 was investigated for the first time using transgenic *Caenorhabditis elegans*. Our results demonstrated that Exendin-4 attenuated the amyloid-β (1-42) (Aβ1-42) toxicity *via* multiple mechanisms, such as depressing its expression on protein and mRNA and reducing Aβ (1-42) accumulation. Exendin-4 at 0.5 mg/ml had been shown to extend life by 34.39% in CL4176 and delay the onset of paralysis in CL4176 and CL2006 which were increased by 8.18 and 8.02%, respectively. With the treatment of Exendin-4, the nuclear translocation of DAF-16 in the transgenic nematode TJ356 was enhanced. Superoxide dismutase-3 (SOD-3), as a downstream target gene regulated by DAF-16, was upregulated on mRNA level and activity. The reactive oxygen species (ROS) level was decreased. In contrast, we observed that the ability of Exendin-4 to regulate SOD was decreased in CL4176 worms with the DAF-16 gene silenced. The activity of SOD and the mRNA level of sod-3 were downregulated by 30.45 and 43.13%, respectively. Taken together, Exendin-4 attenuated Aβ (1-42) toxicity in the *C. elegans* model of AD *via* decreasing the expression and the accumulation of Aβ (1-42). Exendin-4 exhibited the ability of antioxidant stress through DAF-16. With continuous research, Exendin-4 would become a potential therapeutic strategy for treating AD.

## Introduction

As a chronic neurodegenerative disease, Alzheimer's disease (AD) poses a serious threat to individuals' health. Despite years of massive investigation, the pathogenic mechanism of AD is still in the stage of hypothesis theories, such as the hyperphosphorylated tau protein hypothesis, amyloid toxicity hypothesis, oxidative stress hypothesis, and acetylcholine hypothesis. However, the amyloid toxicity hypothesis is widely accepted. Amyloid-β peptides (Aβ1-40 and Aβ1-42) are produced due to aberrant cleavage of the amyloid precursor protein (APP), which accumulates on the neuron. Furthermore, Aβ (1-42), as a mediator of oxidative stress, has been proposed to play a central role in the pathogenesis of AD (Butterfield and Boyd-Kimball, 2004), such as decreasing the levels of superoxide dismutase (SOD) and increasing the levels of MDA and reactive oxygen species (ROS) (Wang et al., 2021). As a target, amyloid is often applied to drug screening and clinical diagnosis.

The data of epidemiological investigation suggest that the correlation between type 2 diabetes mellitus (T2DM) and AD is highly significant. Diabetic patients are twice as likely to suffer from AD as the normal population. AD and T2DM always share common pathophysiological symptoms (Caberlotto et al., 2019), such as hyperglycemia, hypercholesterolemia, and insulin signaling dysfunction.

Thus, it has been strongly suggested that some drugs treated with T2DM may have therapeutic potential in AD (Sebastiao et al., 2014). Exendin-4, as a glucagon-like peptide-1 (GLP-1) receptor agonist, has been approved for the treatment of T2DM. However, the strong preclinical evidence suggests that Exendin-4 is neuroprotective in AD (Mullins et al., 2019). For instance, Exendin-4 appears to prevent the hyperphosphorylation of AD-associated tau protein due to the increased insulin signaling pathway in the brain (Xu et al., 2015). Exendin-4 significantly attenuated Aβ-induced memory deficits in the Morris water maze and Y-maze test (Garabadu and Verma, 2019).

Many kinds of transgenic *Caenorhabditis elegans* models (Wu and Luo, 2005) of AD had been used to research the pathogenesis of AD (Link, 2006) due to their short lifetime and progressive paralysis phenotype. Moreover, the transgenic *C. elegans*, such as CL4176 (Wu and Luo, 2005; Zhang et al., 2016) and CL2006 (Smith and Luo, 2003), promotes the onset of the ROS, and it is similar to the onset of ROS observed in patients with AD, which is consistent with the amyloid cascade hypothesis on oxidative stress. Oxidative stress is extensive in the AD brain. Aβ (1-42) has been shown to induce oxidative stress and neurotoxicity *in vitro* and *in vivo*. Therefore, transgenic *C. elegans* strains accelerate the pace of understanding the mechanisms of Aβ (1-42) toxicity in biological systems. At the same time, they can be used for anti-AD drug screening *in vivo* (Ewald and Li, 2010; Wolozin et al., 2011).

Herewith, we mainly employed transgenic *C. elegans* as the model to investigate the neuroprotective effects of Exendin-4. Our conclusion presents that Exendin-4 develops its neuroprotective effect by reducing the accumulation and expression of Aβ (1-42) directly. Exendin-4 also showed the excellent performance of antioxidant stress *via* the transcription factors DAF-16.

## Materials and methods

### *C. Elegans* strains and maintenance

The wild-type N2 worms and the transgenic worms CL4176 {smg-1ts [myo-3/Aβ1–42 long 3′-untranslated region (UTR)]} were obtained from Caenorhabditis Genetics Center (University of Minnesota, Minneapolis, MN). TJ356 [daf-16p::daf-16a/b::GFP + rol-6], CL2006 {dvIs2 [pCL12(unc-54/human Aβ1–42 minigene) + pRF4]}, the transgenic CF1553 {muIs84 [(pAD76) sod-3p::GFP + rol-6(su1006)]}, and *Escherichia coli* were kindly donated by Dr. Liping Wang. CL4176 was maintained at 16 °C. The wild-type N2, CL2006, CF1553, and TJ356 were maintained at 20 °C.

All worms were on a solid nematode growth medium (NGM) seeded with live *E. coli* (OP50) as a food source.

### Brood size and body length assays

The wild-type N2 worms were used in brood size and body length assays. For the brood size assay, each worm (L4 stage) was transferred onto NGM plates treated with different concentrations of Exendin-4 (0.1, 0.3, 0.5, and 1.2 mg/ml). The worms were cultivated at 20 °C. The number of all eggs that it hatched was recorded (Rangsinth et al., 2019).

For the body length assay, worms (L4 stage) were transferred onto different NGM plates treated with different concentrations of Exendin-4 (0.1, 0.3, 0.5, and 1.2 mg/ml) and cultured at 20 °C. Adult day-1 worms were paralyzed using 0.1% sodium azide and mounted on a glass slide. At least thirty worms per group were imaged using light microscopes (Leica, DM3000 LED, Germany) and measured using the Leica Application Suite version 4.12 software (Leica, Germany).

### Paralysis assays

We synchronized the transgenic *C. elegans* strain CL4176 on different concentrations of Exendin-4 (0 mg/ml, 0.02 mg/ml, 0.1 mg/ml, 0.3 mg/ml, 0.5 mg/ml, and 1.2 mg/ml) and caffeine (1.2 mg/ml) treated plates at 16 °C for 48 h. Then, worms (L2 stage) were transferred from 16 °C to 23 °C. After cultivating 40 h at 23 °C, the paralyzed worms had been counted at 2 h intervals until all the worms were paralyzed (Dostal and Link, 2010; Takahashi et al., 2014). The principle of paralysis is stipulated that they moved their heads only or failed to move their bodies by touching stimuli.

### Life span assays

To obtain the synchronized worms, gravid adults (L1 stage) of CL4176 were raised on different concentrations of Exendin-4 (0 mg/ml, 0.02 mg/ml, 0.1 mg/ml, 0.3 mg/ml, and 0.5 mg/ml)-treated plates to lay eggs for 2–3 h. Generally, 280 worms should be promised in one plate. After 48 h of synchronization, Aβ (1-42) expression was induced by upshifting the temperature to 23 °C. We continued to collect the data for 20 days. All life span assays were preceded independently at least three times. Worms that were missing, attaching to walls or worm bags, were not included during the life statistics.

### Western blotting

Worms were treated with Exendin-4 (0.5 mg/ml) and induced for 44 h at 23 °C. Worms (L4 stage) were harvested and centrifuged at 12,000 rpm for 10 min. The pellets were resuspended and sonicated after freeze-thawed as described in the WormBook (http://www.wormbook.org). The gel was transferred onto polyvinylidene fluoride (PVDF) membranes. The monoclonal antibody (ab201060, ABCam, UK) was used to detect Aβ (1-42). Experiments were repeated independently for three times.

### Staining of Aβ

Worms (L2 stage) were transferred from 16 °C to 23 °C to cultivate for 40 h after synchronization. A total of 100 worms (L4 stage) were picked up and washed with M9 buffer. After centrifugation, worms were fixed with 150 μl of 4%paraformaldehyde/PBS buffer (pH = 7.4) at 4 °C for 24 h. Fixed worms were treated with 150 μl 5% β-mercaptoethanol, 1% Triton X-100, and 125 mM Tris pH 7.4, at 37 °C for 24 h. Treated worms were washed with PBS buffer three times, and 200 μl of 0.125% thioflavin T (Sigma) in 50% ethanol was added for 90 s and destained with 500 μl of 50% ethanol until the suspension became transparent. Worms were resuspended with 100 μl M9 buffer. The animals were finally transferred to slides using a drop of glycerol, and fluorescence images were acquired by confocal laser scanning fluorescence microscopy (CarlZeiss LSM710, Germany).

### ROS assays

Age-synchronized *C. elegans* (more than 30 eggs per plate) were transferred to NGM plates containing vehicle or 0.5 mg/ml Exendin-4 and incubated for 48 h at 16 °C. After incubation at 23 °C for 44 h, ROS was determined based on published studies (Wu et al., 2012). The examined nematodes were transferred to 0.5 ml of M9 buffer containing 5 μM CM-H2DCFDA (D6883, Sigma-Aldrich, USA) and preincubated for 3 h at 20 °C. Later, nematodes were mounted on 2 % agar pads and examined using a fluorescence microscope (Nikon, SMZ 18, Japan) at 495 nm of excitation wavelength and 537 nm of emission filter. More than 30 animals were counted for the statistical analysis. The relative fluorescence intensities of the worm were semi-quantified using the ImageJ software. The semi-quantified ROS was expressed as relative fluorescence units (RFU). Three replicates were performed.

### SOD activity assays

The transgenic *C. elegans* strain CL4176 was synchronized on 0.5 mg/ml Exendin-4 treated and untreated plates at 16 °C for 48 h, respectively. Then, the worms (L2 stage) were cultivated for another 40 h at 23 °C. A total of 200 worms (L4 stage) were collected and centrifuged with M9 buffer. The pellets were disrupted according to the Western blotting assay protocol. After protein quantification using bicinchoninic acid (BCA) assay, the analysis of SOD activity was proceeded using nitrotetrazolium blue chloride (NBT) method (Total Superoxide Dismutase Assay Kit with NBT, #S0109, Beyotime Biotechnology, China). Four replicates were performed.

### Real-time PCR analysis

Worms were treated according to the Western blotting protocol. The worms (L4 stage) were collected by washing the plate with 1 ml M9 buffer. The pellets were washed three times by centrifugation at 25,000 rpm for 5 min. The worms were freeze-thawed and transferred directly into 1 ml of TRIzol reagent (Thermo Fisher Scientific, Shanghai, China). The total nematode RNA was extracted first and measured using a Nanophotometer (IMPLEN N60, German). The cDNAs were synthesized using the Transcription Kit (Promega, A5000, USA). The expression of genes was determined by real-time PCR performed on 7500 Real-Time PCR System (ABI) with SYBR Green real-time PCR kit (Roche). According to the instruction of the real-time PCR kit, the cycling conditions were as follows: (1) holding stage at 50 °C for 2 min; (2) holding stage at 95 °C for 10 min; (3) cycling stage at 95 °C for 15 s, at 55 °C for 1 min; the number of cycling for 40 cycles; and (4) melt curve stage at 95 °C for 10 s, at 60 °C for 1 min, at 95 °C for 10 s. The relative levels of gene expression were calculated using the 2 ^−Δ*ΔCT*^ method using the gene actin-1 as the internal control. The experiment was repeated in triplicate. Primers are listed in Table 1.

**Table 1:** Lists of primers.

**Gene**	**Forward primer**	**Reverse primer**
actin-1	5′ AAGACCACGTCATCAAGG3′	5′TTCTCCATATCATCCCAGTT3′
daf-16	5′GCGAATCGGTTCCAGCAATTCCAA3′	5′ATCCACGGACACTGTTCAACTCGT3′
sod-3	TATTAAGCGCGACTTCGGTTCCCT	CGTGCTCCCAAACGTCAATTCCAA3
Aβ (1-42)	CCGACATGACTCAGGATATGAAGT	CACCATGAGTCCAATGATTGCA3

### DAF-16 nuclear translocation analysis

Age-synchronized nematodes of the transgenic strain TJ356 stably expressing a DAF-16::GFP fusion protein were treated with Exendin-4 (0.5 mg/ml) and kept at 20 °C for 48 h. Later, the worms (L4 stage) were anesthetized with 0.1% sodium and transferred to slides using a drop of glycerol. The subcellular DAF-16 distribution among twenty worms per group was analyzed using a Nikon SMZ 1500 fluorescence microscope. The experiments were performed more than 3 times. The amount of DAF-16::GFP was analyzed using the ImageJ software.

### The *C. Elegans* RNA interference assay

Worms (L1 stage) were treated with 0.5 mg/ml Exendin-4. DAF-16 was knocked down by feeding the *C. elegans* CL4176 with *E. coli* strain R13H8.1 bacteria carrying daf-16 dsRNA. Worms fed with R13H8.1 bacteria with the empty vector L4440 were used as negative controls. Notably, 1 mM isopropyl β-D-1-thiogalactopyranoside (IPTG) was used on NGM plates. CL4176 was maintained at 16 °C after synchronization. After 48 h, more than 50 worms (L2 stage) were cultured for another 40 h at 23 °C. Then, the process of paralysis assays was carried out. The experiments were performed more than 3 times.

### Statistical analysis

Statistical analyses of differences between groups in the paralysis assays and life span assay were performed using the log-rank test. Data other than paralysis and life span were analyzed using Student's *t*-test. Results were expressed as the mean ± standard deviation of experiments repeated independently for three times. The GraphPad Prism software 5.0 (GraphPad, La Jolla, CA, United States) was used for statistical analyses. The difference in statistical data is shown with *p*-value; *p* < 0.05 (^*^), *p* < 0.01 (^**^), and *p* < 0.001 (^***^) were regarded as significant.

## Results

### Effects of exendin-4 on the development and reproduction of *C. Elegans*

**Figure 1:**
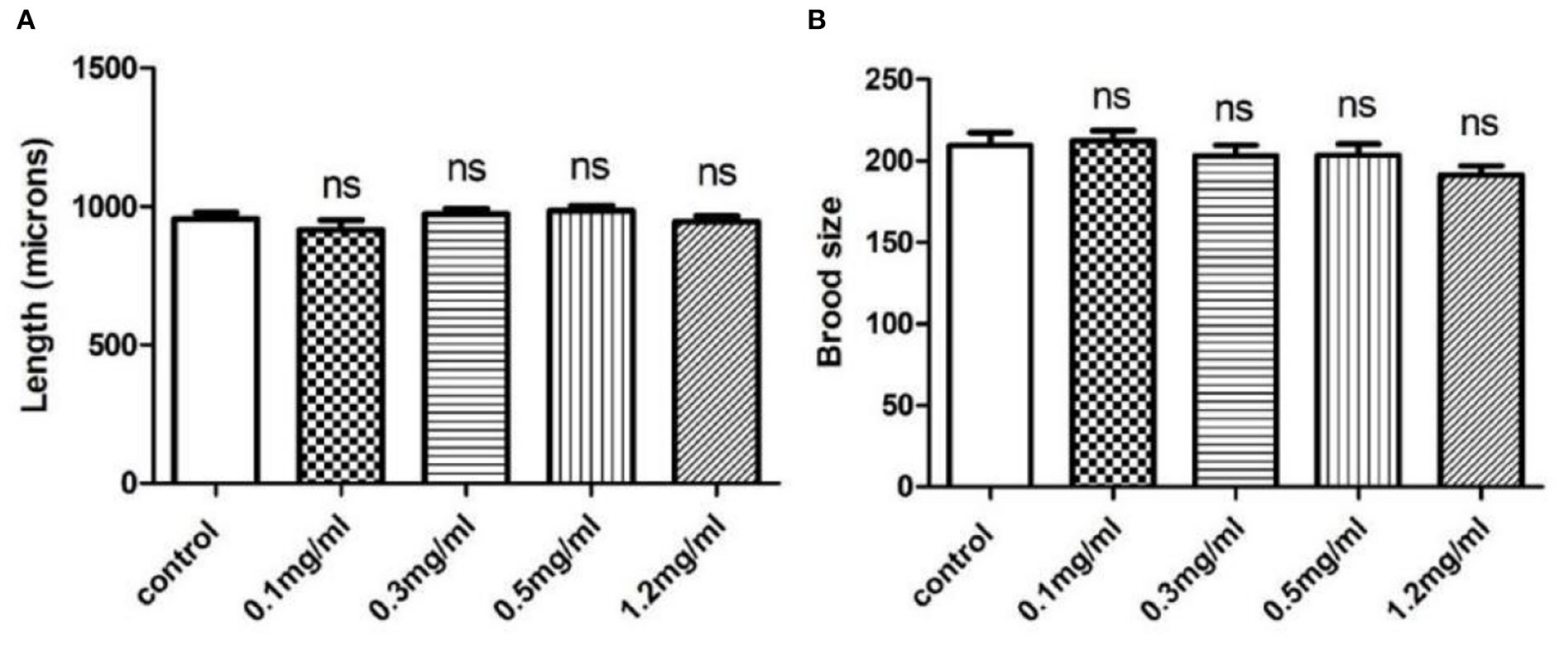
The effects of Exendin-4 on wild-type N2 *Caenorhabditis elegans*. **(A)** Body length assay and **(B)** brood size assay. There was no significant difference in the treatment groups on brood size and body length. Values are mean ± SEM of experiments repeated independently at least for three times.

To investigate the toxicity of Exendin-4, we performed body length and brood size assays to monitor the development and fertility rate of wild-type N2 worms, respectively. The data showed that Exendin-4 with 0.1, 0.3, 0.5, and 1.2 mg/ml has no toxic effects on nematodes in terms of spawning and body length (Figures 1A,B).

### Exendin-4 delays the progression of paralysis in AD worms

**Figure 2:**
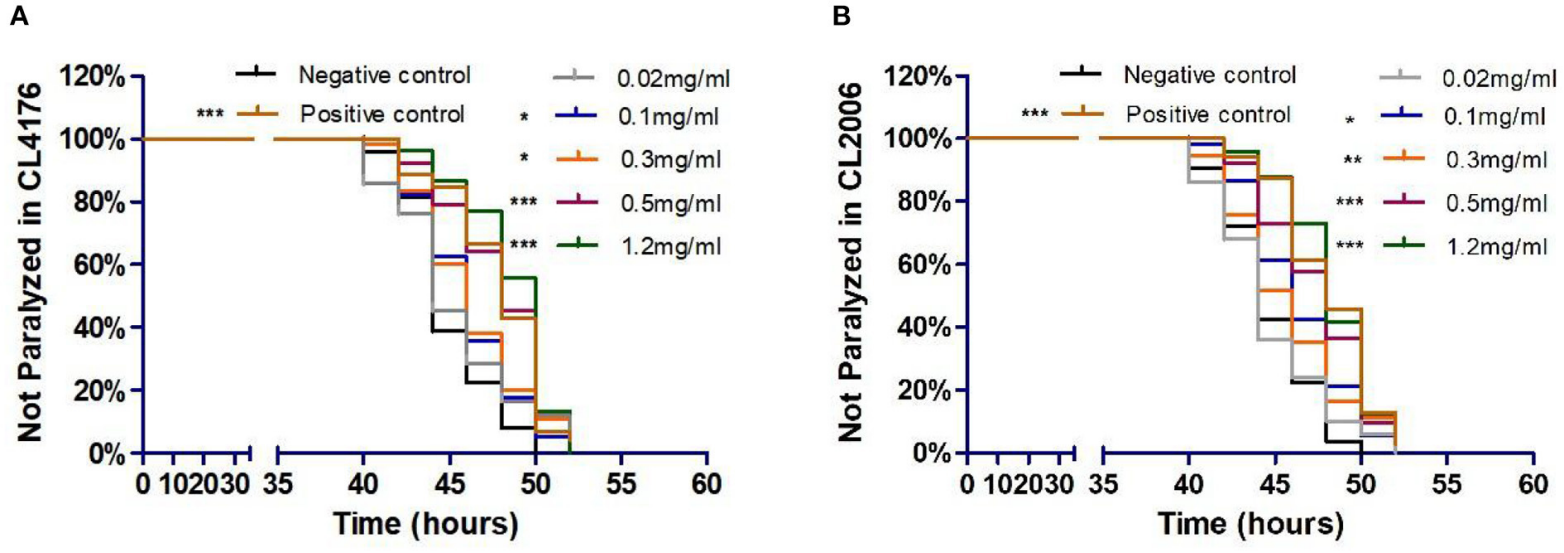
Exendin-4 alleviated Aβ-induced paralysis in transgenic *C. elegans* strains CL4176 and CL2006. **(A)** In CL4176. **(B)** In CL2006. Curves show the process of Aβ-induced paralysis in transgenic *C. elegans* treated with a vehicle control (H_2_O) or different concentrations of Exendin-4 (0.02 mg/ml, 0.1 mg/ml, 0.3 mg/ml, 0.5 mg/ml, and 1.2 mg/ml). Caffeine (1.2 mg/ml) is positive control. The onset of paralysis was prolonged in CL4176 and CL2006 worms in a dose-dependent manner (at least 40 worms were tested in each group, and the experiments were repeated independently for three times).

To investigate the potential influence of Exendin-4 on the progression of paralysis induced by A β(1-42), we treated CL4176 and CL2006, respectively. The onset of paralysis was dramatically delayed in a dose-dependent manner after treatment of Exendin-4 (Figures 2A,B). The low dose of Exendin-4 (0.02 mg/ml) cannot slow down the process of paralysis significantly. Notably, 1.2 mg/ml of Exendin-4 displayed the best effect among all groups. The mean paralysis time was increased by 8.18% (CL4176) and 8.02% (CL2006), respectively. In CL4176, the duration of paralysis in the 1.2 mg/ml Exendin-4 group was prolonged by 3.67 h. The average paralysis time was 0.7 h longer than that of the positive control (Supplementary Table 1). Exendin-4 showed better performance than the positive control. In CL2006, the duration of paralysis in the 1.2 mg/ml Exendin-4 group was prolonged by 3.58 h. The average paralysis time was 0.11 h longer than that of the positive control (Supplementary Table 2). Combined with the experimental results in the two mutant nematodes, these observations suggested that Exendin-4 may have the potential to relieve the paralysis caused by Aβ (1-42).

### Exendin-4 increases life span in AD worms

**Figure 3:**
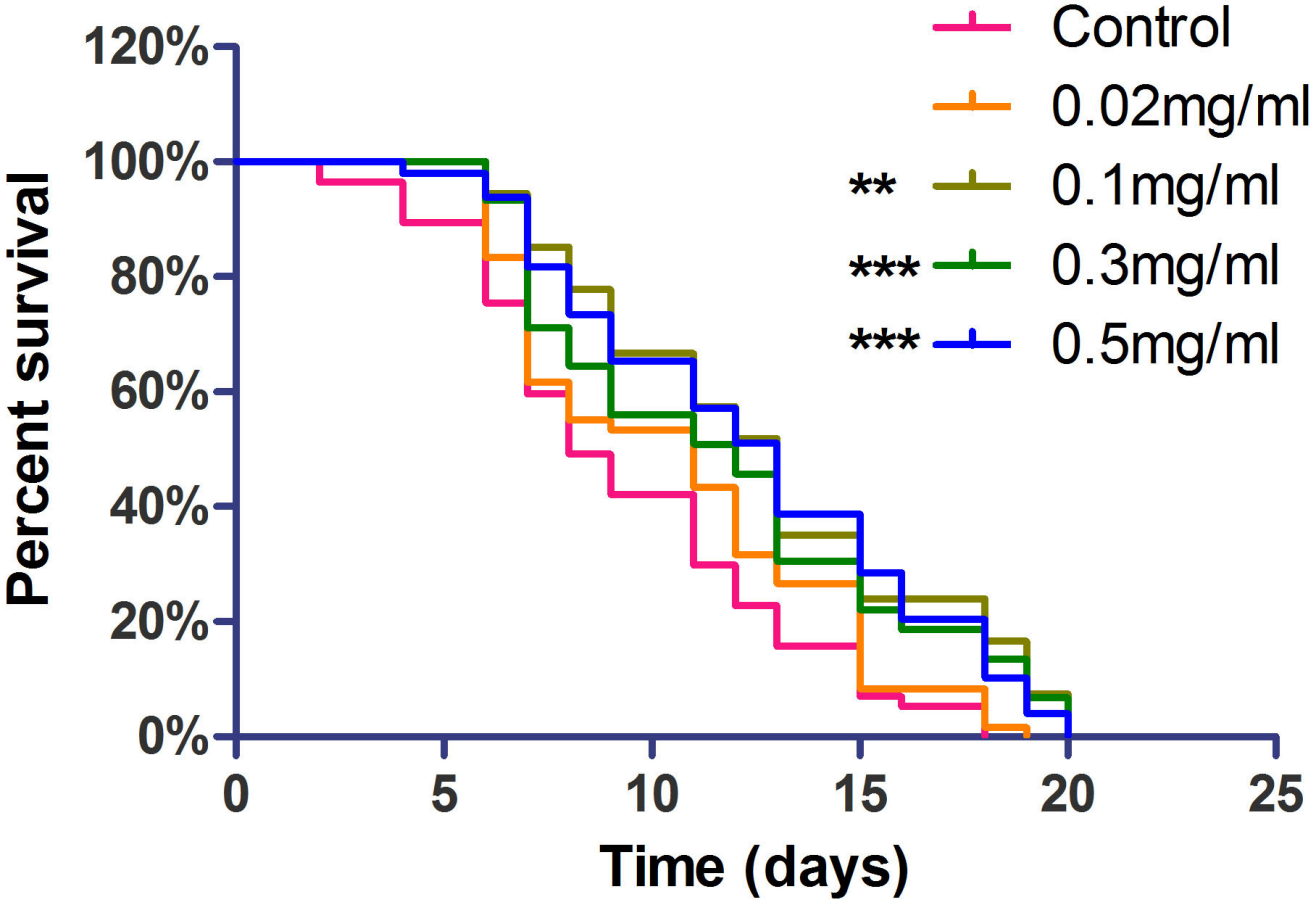
The life span assay administrated Exendin-4 in transgenic *C. elegans* strain CL4176. CL4176 was treated with a vehicle control (H_2_O) or different concentrations of Exendin-4 (0.02 mg/ml, 0.1 mg/ml, 0.3 mg/ml, and 0.5 mg/ml). The median survival of worms treated with 0.5 mg/ml Exendin-4 was 13 days, which had a significant expansion compared with 8 days of control worms (*p* < 0.001). The experiment was repeated independently for three times.

The life span assay was examined under different Exendin-4 concentrations (0.02, 0.1, 0.3, and 0.5 mg/ml). We collected the data for 20 days. Exendin-4 treatment showed a significant increase in the life span in a dose-dependent manner (Figure 3). Median survival time was prolonged in each treated group. Notably, 0.3 mg/ml and 0.5 mg/ml of Exendin-4 displayed remarkable performance in extending life span. They almost increased the median survival time to 13 days. Compared with the control group, lifetime was increased by 32.05 and 34.39%, respectively (Supplementary Table 3). Results implied that Exendin-4 treatment attenuated the harmful effect of longevity induced by Aβ (1-42) toxicity in worms.

### Exendin-4 reduced the levels of Aβ (1-42) in AD worms

**Figure 4:**
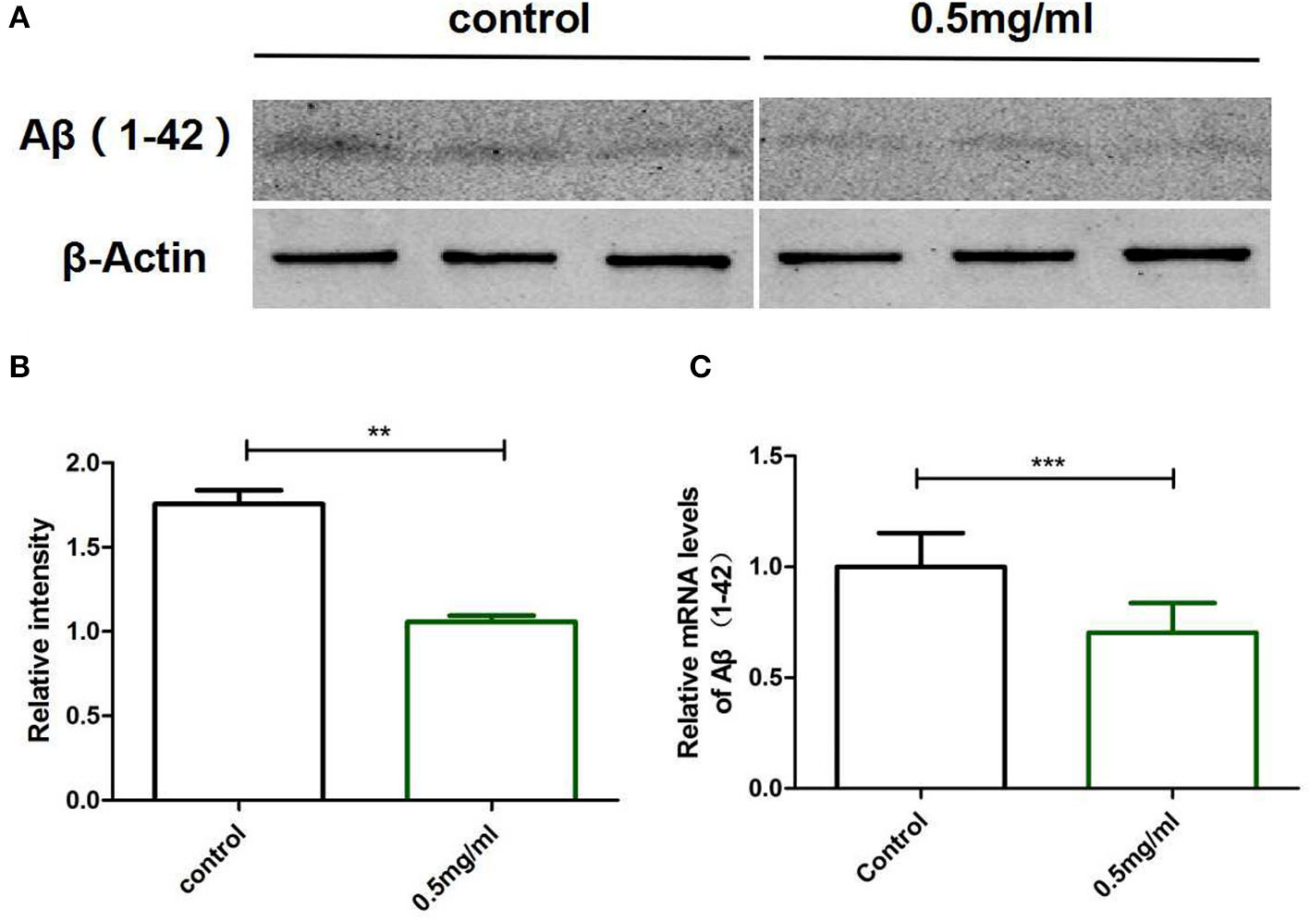
Exendin-4 attenuated the protein and mRNA levels of Aβ (1-42) in *C. elegans* CL4176. **(A)** Western blotting assays of experiments repeated independently for three times are shown. Control groups were not treated with Exendin-4. The test groups were treated with 0.5 mg/ml of Exendin-4. **(B)** The relative intensity of Aβ (1-42) expression was analyzed using the ImageJ software. After treatment with 0.5 mg/ml Exendin-4, the protein expression level was reduced by 39.78%. *p* < 0.01 (^**^).**(C)** The mRNA levels of Aβ (1-42) were analyzed by real-time PCR. mRNA levels were reduced by 29.67%. *p* < 0.001 (***).

**Figure 5:**
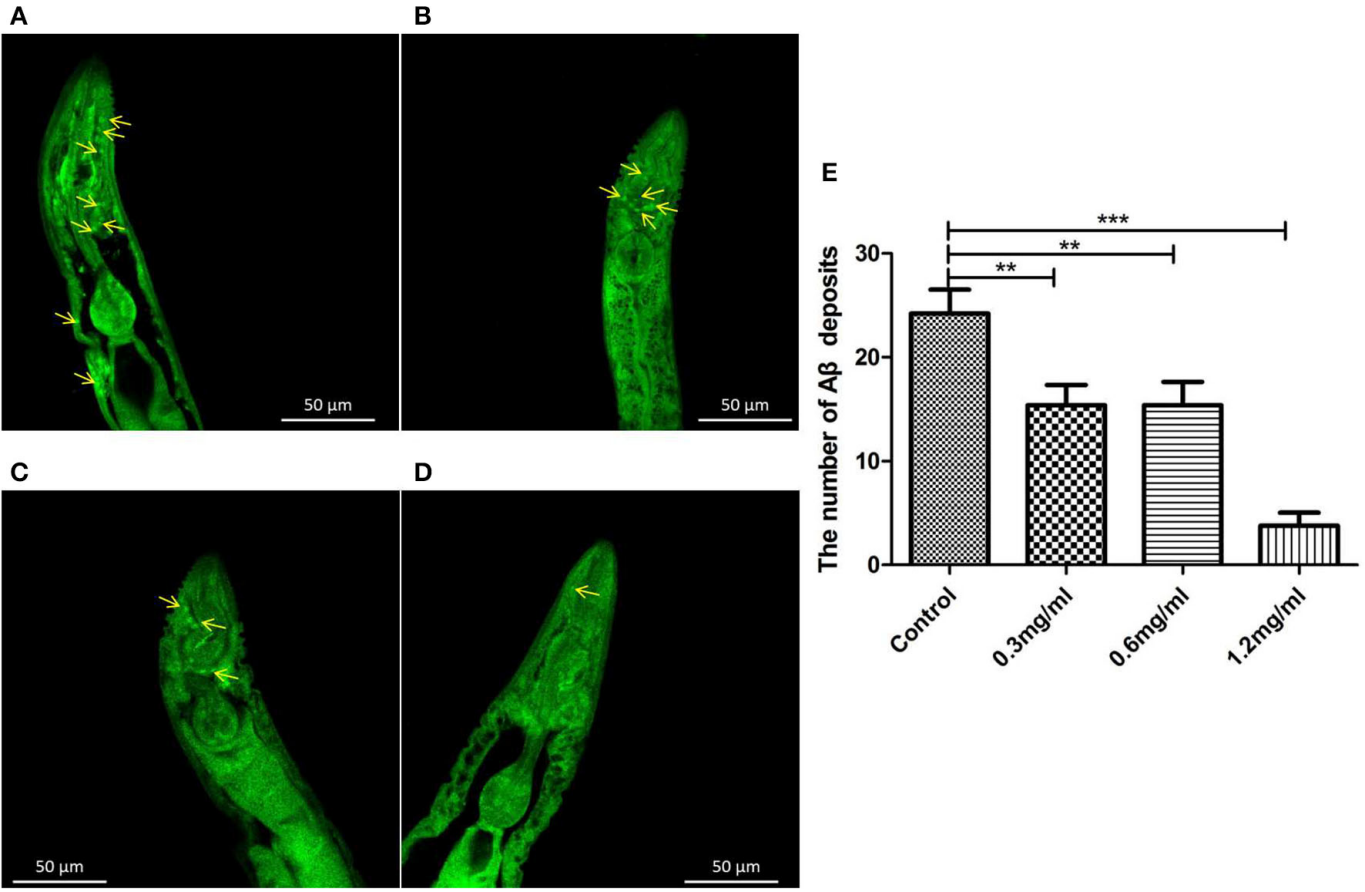
Aβ (1-42) accumulation assay with thioflavin T staining in transgenic *C. elegans* CL4176. **(A)** Control groups were not treated with Exendin-4. Aβ (1-42) deposits stained with thioflavin-T show abundant fluorescence patches near the pharynx in transgenic worm CL4176. **(B–D)** Transgenic worms fed with 0.3 mg/ml, 0.6 mg/ml, and 1.2 mg/ml Exendin-4. They show a significant reduction in the deposition of Aβ (1-42) in a dose-dependent way. The yellow arrows show the sites of Aβ (1-42). **(E)** The number of Aβ (1-42) patches in different groups. *p* < 0.01 (**) and *p* < 0.001 (***).

To clarify why Exendin-4 can relieve paralysis in CL4176, we monitored the Aβ (1-42) levels on protein and mRNA. All worms were collected after being induced to paralysis for 44 h, and parallel populations were processed for real-time PCR and the Western blotting assay. The group treated with 0.5 mg/ml Exendin-4 resulted in a 39.78% decrease in the protein expression of Aβ (1-42) (Figures 4A,B) and a significant reduction by 29.67% in the mRNA level of Aβ (1-42) (Figure 4C). Thus, the ability of Exendin-4 to delay paralysis is directly demonstrated by downregulating the protein and mRNA levels of Aβ (1-42). We detected the change of Aβ (1-42) accumulation with a thioflavin T staining assay. We focused on the area near the pharynx of *C. elegans* CL4176 to count the number of deposits. Lots of Aβ (1-42) deposits appeared after being induced (Figures 5A–D). Instead, worms treated with Exendin-4 displayed less Aβ (1-42) deposits in a dose-dependent manner (Figure 5E). Exendin-4 possessed the positive effect to inhibit the accumulation of Aβ (1-42).

### Exendin-4 increases oxidative stress resistance in AD worms

The ROS will be increased by triggering the expression of Aβ (1-42). The increased production of ROS associated with age and neurotransmission in neurons leads to cognitive dysfunction in AD.

To test the antioxidant effect of Exendin-4, two kinds of strains (CL2006 and CL4176) were treated with 0.5 mg/ml of Exendin-4.

**Figure 6:**
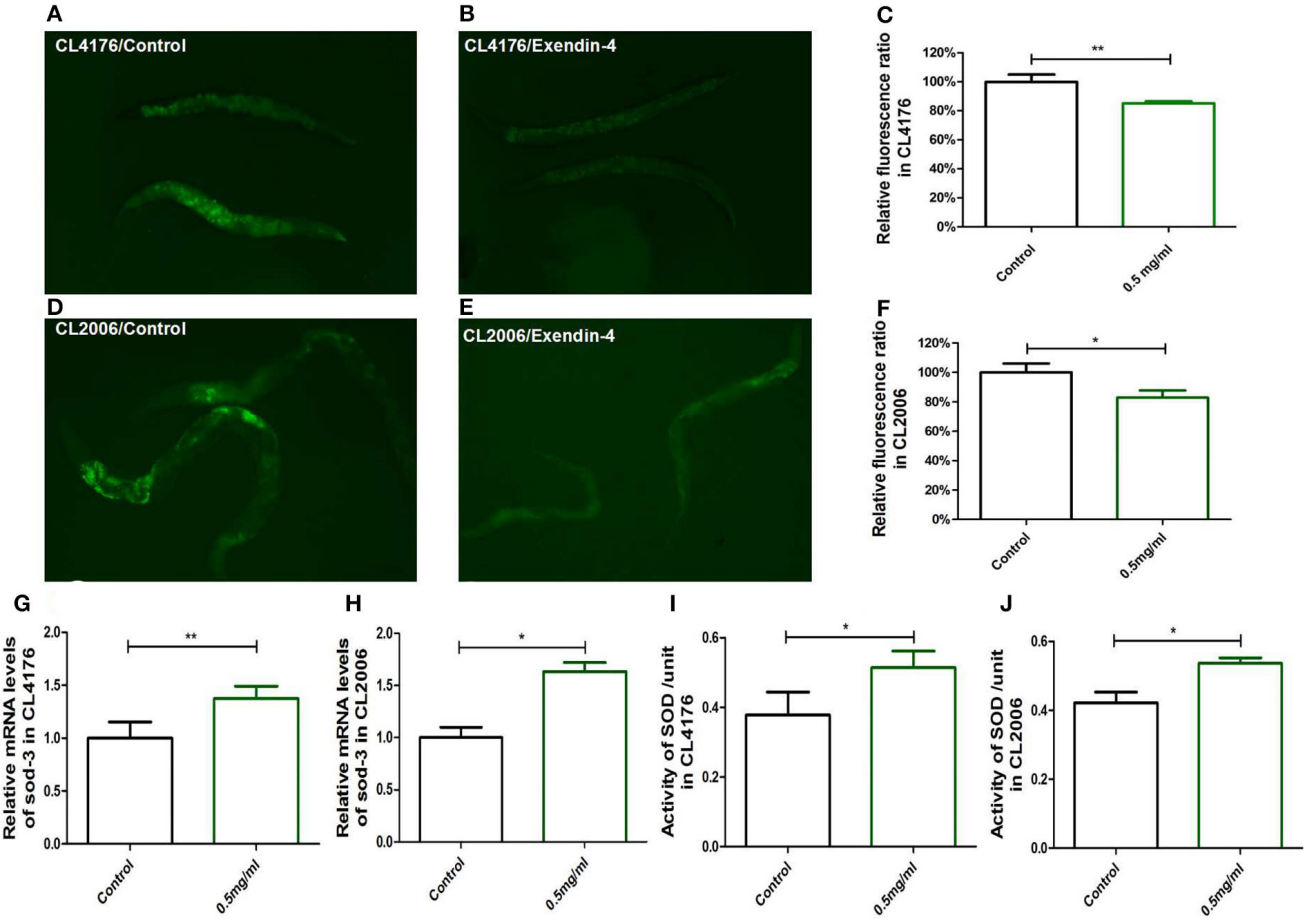
The effect of antioxidant stress of Exendin-4 in *C. elegans* CL4176 and CL2006. ROS was analyzed using CM-H2DCFDA. Photographs were taken using a fluorescence microscope at 495 nm of excitation wavelength and 537 nm of emission filter. **(A,D)** The untreated CL4176 and CL2006. **(B,E)** The treated CL4176 and CL2006 with 0.5 mg/ml Exendin-4. **(C,F)** The fluorescence intensity analyzed using the ImageJ software. **(G,H)** The mRNA levels of superoxide dismutase-3 (sod-3). **(I,J)** The activity of sod-3. All the experiments were repeated independently for three times. *p* < 0.05 (*), *p* < 0.01 (**).

Reactive oxygen species was measured using the CM-H2-DCFDA method. The worms treated with Exendin-4 showed lower production of ROS in CL2006 and CL4176 (Figures 6A,B,D,E). The fluorescence intensity was analyzed using the ImageJ software. A volume of 0.5 mg/ml Exendin-4 decreased ROS level by 13.57% (*p* < 0.01) and 17.22% (*p* < 0.05) in CL4176 and CL2006, respectively (Figures 6C,F). A volume of 0.5 mg/ml Exendin-4 improved the mRNA levels of sod-3 by 37.33% (*p* < 0.01) and 63.33% (*p* < 0.05) in CL4176 and CL2006, respectively (Figures 6G,H). A volume of 0.5 mg/ml Exendin-4 improved the activity of sod-3 by 36.04% (*p* < 0.05) and 27.11% (*p* < 0.05) in CL4176 and CL2006, respectively (Figures 6I,J).

We have a primary judgment that Exendin-4 is provided with delaying the process of AD induced by Aβ toxicity through its antioxidative stress activity.

### DAF-16 was essential to the neuroprotective effect of exendin-4

**Figure 7:**
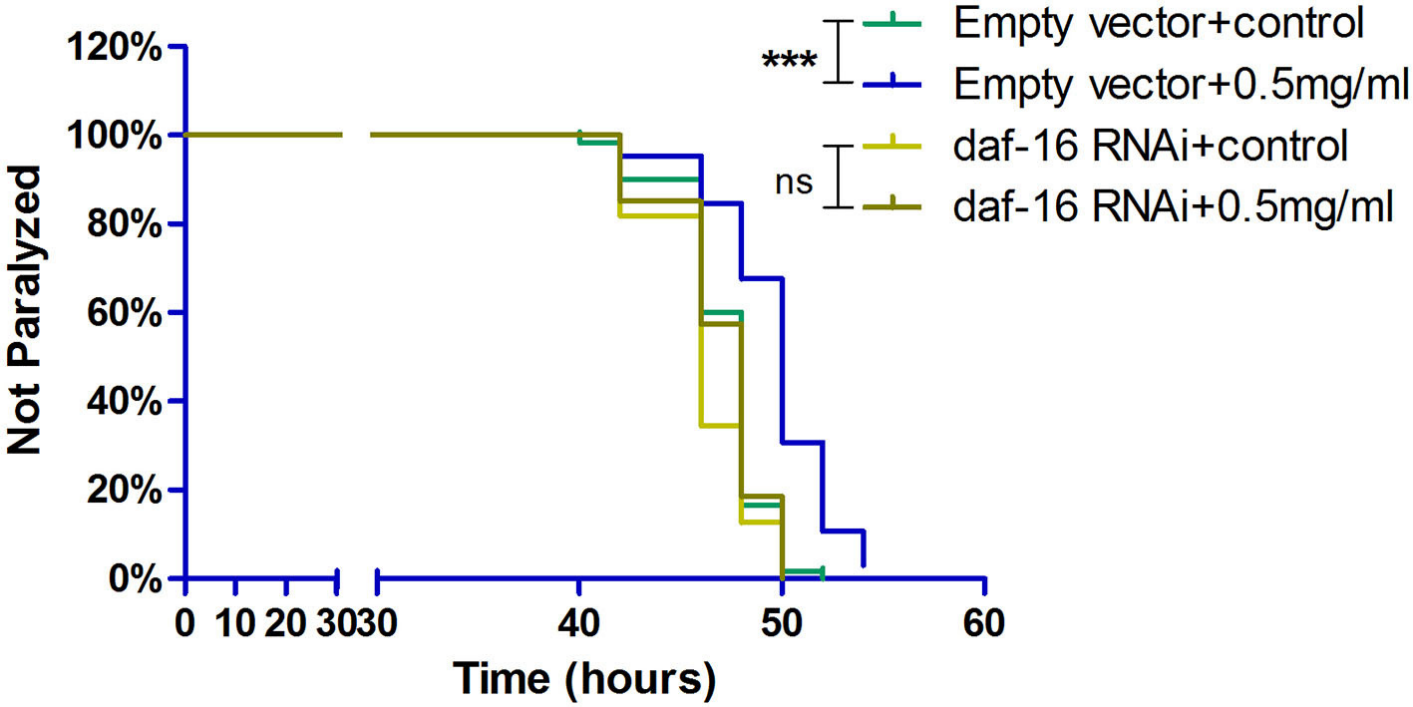
Exendin-4 lost the ability to alleviate Aβ-induced paralysis when the daf-16 gene is knockout in transgenic *C. elegans* strain CL4176. DAF-16 expression was knocked down by feeding the *C. elegans* CL4176 with *Escherichia coli* strain R13H8.1 bacteria carrying daf-16 dsRNA. Curves show that the effect of Exendin-4 (0.5 mg/ml) on inhibiting the paralysis rate of *C. elegans* vanished after daf-16 gene knockout. (At least 50 worms were tested in each group, and the experiments were repeated independently for three times.).

DAF-16, which is a major regulator in the insulin/insulin-like growth factor 1 signaling (IIS) pathway, integrates signals from upstream pathways to elicit transcriptional changes in many genes involved in aging, development, stress, metabolism, and immunity. Exendin-4 has a profound effect on the insulin signaling pathway to prevent the process of AD (Xu et al., 2015; Yang et al., 2016). Hence, we investigated whether Exendin-4 can delay the process of paralysis in worms when daf-16 was knocked down using RNA interference (RNAi) (Supplementary Figure 2). Exendin-4 at 0.5 mg/ml lost its ability to delay Aβ-induced paralysis significantly when daf-16 of the worms was knockout (Figure 7).

**Figure 8:**
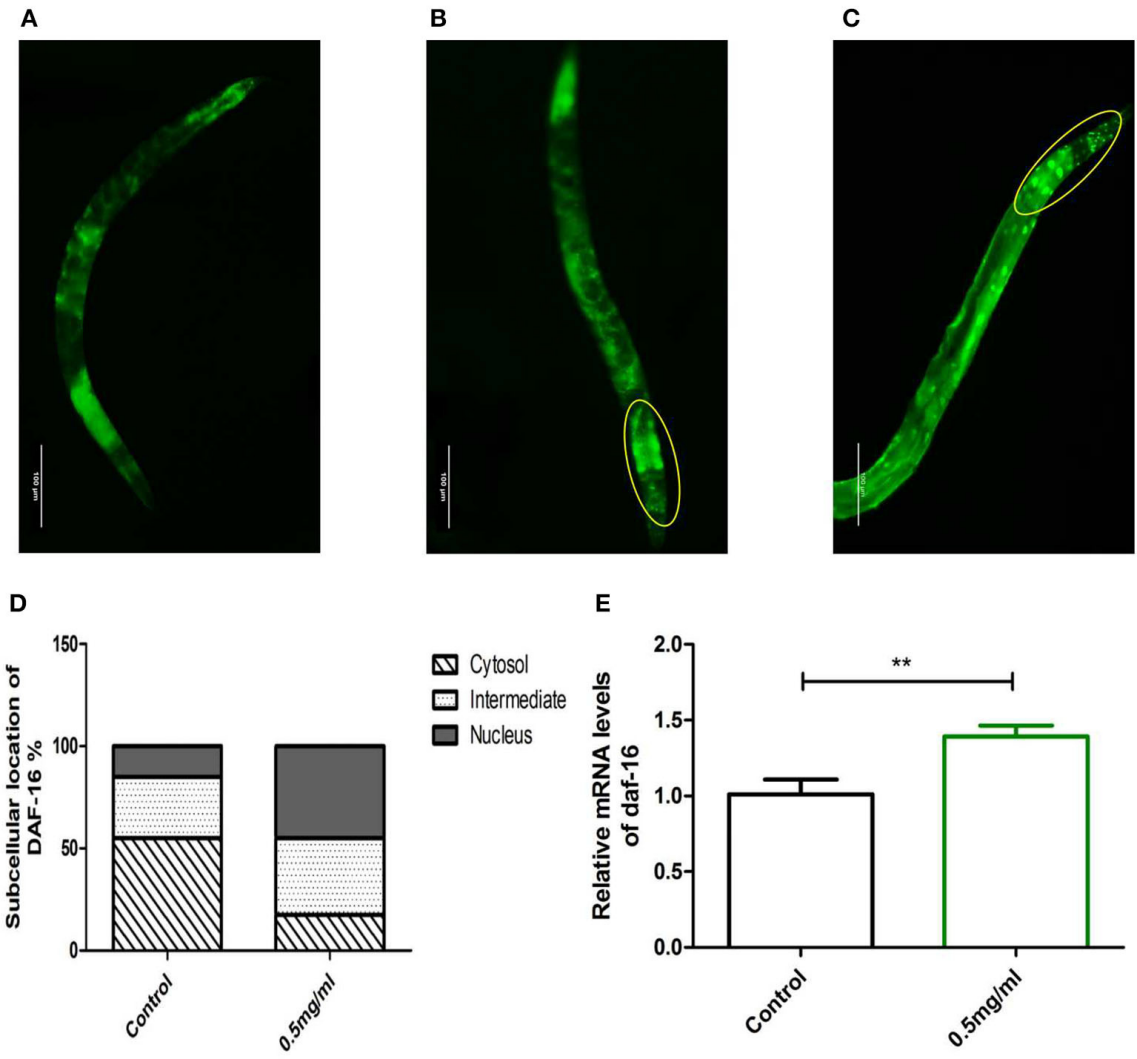
The effect of Exendin-4 on DAF-16. The subcellular distribution of DAF-16 was examined in approximately 20 worms for each group by fluorescence microscopy. **(A)** Cytosol, **(B)** intermediate, **(C)** nucleus, and **(D)** the treated worms with 0.5 mg/ml Exendin-4 significantly increase the ratio of nucleus translocation. The histogram represents the ratio of each subcellular distribution in the whole population in each group. **(E)** The mRNA levels of daf-16 were analyzed by real-time PCR. mRNA levels was increased by 69.00% (*p* < 0.001).

Meanwhile, we examined whether Exendin-4 affected the translocation of DAF-16. The transgenic strain TJ356 can stably express a DAF-16::GFP fusion protein. The indication of fluorescence showed that Exendin-4 enhanced the translocation of DAF-16 from the cytoplasm to nuclei. The subcellular distribution includes “cytosol,” “intermediate,” and “nucleus” (Figures 8A–C). The amount of DAF-16 in nuclei was improved obviously by estimating the fluorescence particles using the ImageJ software (Figure 8D). The ratio of nucleus location was increased from 15 to 45% compared with control worms.

However, Exendin-4 was still able to improve the mRNA expression levels of the daf-16 gene (Figure 8E). These findings indicated that DAF-16 was required for the protective effects of Exendin-4 on Aβ (1-42) toxicity.

### Exendin-4 regulates SOD *via* DAF-16

**Figure 9:**
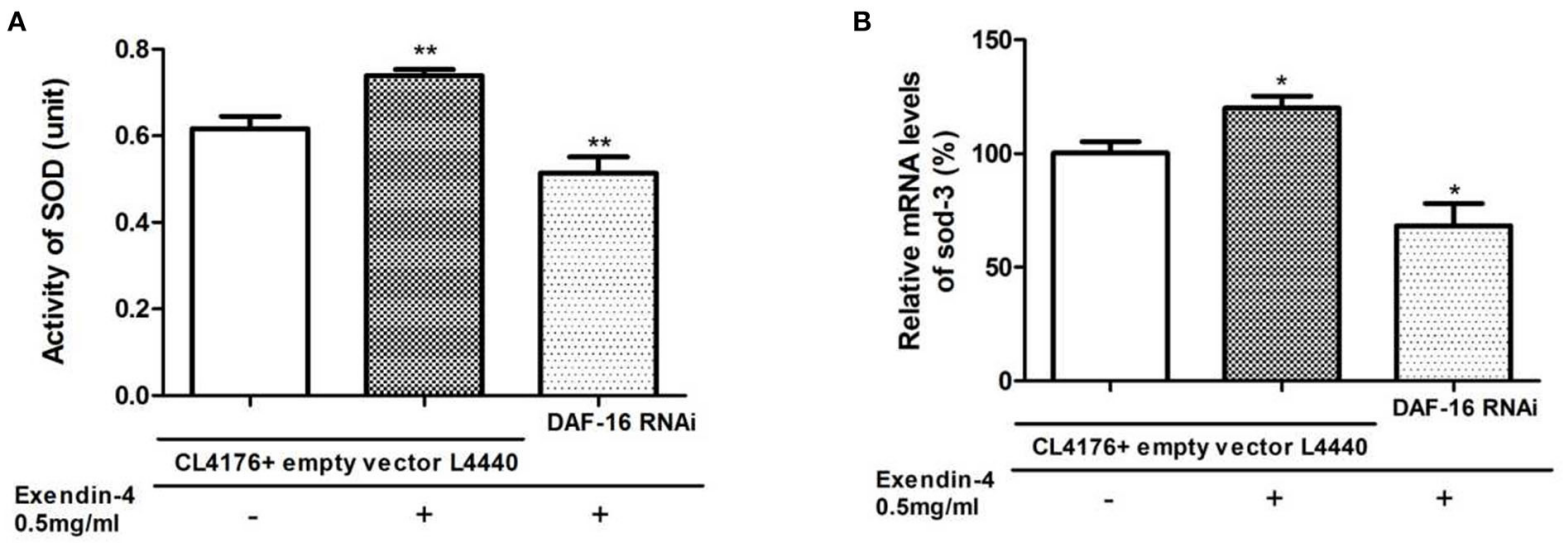
Exendin-4 regulates SOD *via* DAF-16. CL4176 worms with empty vector L4440 as control were treated with or without 0.5 mg/ml Exendin-4. CL4176 worms were knocked down the daf-16 gene by RNAi and treated with 0.5 mg/ml Exendin-4. **(A)** The total SOD activity in worms was investigated using the NBT method. Compared with the control group treated with Exendin-4, the SOD activity was decreased by 30.45% after knocking down the daf-16 gene (*p* < 0.01). **(B)** Compared with the control group treated with Exendin-4, the mRNA levels of sod-3 were decreased by 43.13% after knocking down the daf-16 gene (*p* < 0.05).

SOD-3 is a typical downstream target regulated by DAF-16. To explore whether Exendin-4 regulates SOD *via* DAF-16, SOD was detected in the activity and mRNA levels after knocking down the gene of DAF-16 by RNAi. We observed that the ability of Exendin-4 to regulate SOD decreased in CL4176 worms with the DAF-16 gene silenced. The activity of SOD and mRNA level of sod-3 was downregulated by 30.45 and 43.13%, respectively (Figures 9A,B). The CL4176 worms with empty vector L4440 were administrated with or without Exendin-4, and the same trends as the above results in Figure 6 were displayed due to the existence of DAF-16. All results indicated that Exendin-4 regulated the SOD at least partially through a DAF-16-based mechanism.

## Discussion

Epidemiological studies showed that diabetes is a high-risk factor for Alzheimer's disease. Common pathophysiological features exist between T2DM and AD, including oxidative stress and insulin resistance. It suggests that effective drugs for T2DM could provide an effective treatment option for AD. Exendin-4 is a GLP-1R agonist approved to treat T2DM. Recently, Exendin-4 has shown neuroprotective effects *in vitro* and *in vivo* model systems (Wang et al., 2015, 2016; Qiu et al., 2016; Rocha-Ferreira et al., 2018). However, the neuroprotective effect of Exendin-4 on AD has never been reported in the *C. elegans* models.

In this research, we investigated the neuroprotective effects of Exendin-4 in *C. elegans* model of AD for the first time. We found that Exendin-4 alleviated Aβ (1-42) toxicity *via* DAF-16.

We utilized transgenic *C. elegans* strains CL4176 and CL2006 induced expressing Aβ (1-42) to imitate the onset of paralysis. Our results indicated that Exendin-4 attenuated the process of paralysis and enhanced life span in a dose-dependent manner.

So far, it has not been reported that Exendin-4 can inhibit the onset of paralysis. In this research, we clarified that Exendin-4 possesses the effect of inhibiting paralysis in *C. elegans* model of AD for the first time.

However, it has also been reported that Exendin-4 possesses the ability to increase life span in Huntington's disease mice (Martin et al., 2012). We have proved again that Exendin-4 has the effect of prolonging life span in *C. elegans* model of AD.

We hypothesized that exendin-4 can inhibit paralysis rates or prolong the life of *C. elegans* due to its ability to attenuate Aβ (1-42) toxicity. The content of Aβ (1-42) is the critical reason for its toxicity.

In previous studies, Exendin-4 mainly focuses on the study of protecting against cascading damage caused by Aβ (1-42), such as inhibiting cell apoptosis (Qiu et al., 2016) and relieving oxidative damage (An et al., 2019). Few studies were focused on how Exendin-4 affects the change of Aβ (1-42) directly. Our results of Western blotting and qPCR testified that Exendin-4 attenuated the content of Aβ (1-42) on protein and mRNA levels directly. Subsequently, Aβ (1-42) aggregation deposits were reduced.

As we all know, ROS played a negative role in longevity (Sanz, 2016; Dilberger et al., 2019; Huang et al., 2021) and paralysis (Tang et al., 2022), and the aggregation of Aβ (1-42) will lead to the accumulation of ROS, which will affect the onset of paralysis and life span (Ewald, 2018; Kalmankar et al., 2021). Our results showed that exendin-4 reduced ROS, which was beneficial to prolong life and improve paralysis.

We traced the effect of SOD. We found that the SOD was improved in its activity and mRNA level. It was a positive effect on decreasing ROS content. This kind of effect is consistent with previous studies that Exendin-4 upregulated the SOD on the activity (Zhou et al., 2016) and gene expression in neuronal cells (Hu et al., 2013; Yasuda et al., 2016).

The antioxidant effect of Exendin-4 has been described in many oxidative stress models induced by different drugs *in vivo* and *in vitro*. Exendin-4 may inhibit lipotoxicity-induced oxidative stress in β-cells (Shen et al., 2020). Exendin-4 alleviates oxidative stress by activating Nrf2/HO-1 in streptozotocin-induced diabetic mice (Fang et al., 2019). This is the first study to report that Exendin-4 alleviates the oxidative stress induced by Aβ (1-42) in *C. elegans*.

As a known nuclear transcription factor, DAF-16 can induce transcriptional regulation of many genes involved in aging, development, and stress. SOD-3, as a downstream target gene, is regulated by DAF-16. To study whether Exendin-4 regulates SOD through DAF-16/FOXO, the nuclear translocation of DAF-16 was detected in transgenic *C. elegans* TJ356 expressed DAF-16::GFP. We found that Exendin-4 promoted the nuclear translocation of DAF-16. The nuclear translocation of DAF-16 contributed to the upregulation of SOD-3, leading to the antioxidant effect of Exendin-4. In contrast, worms knocked down daf-16 gene were treated with Exendin-4. We found that the ability of Exendin-4 to upregulate SOD was reduced.

Meanwhile, Exendin-4 lost the ability to alleviate the process of paralysis after silencing the daf-16 gene. These results indicated that DAF-16 played a pivotal role in alleviating Aβ (1-42) toxicity when Exendin-4 exerted its neuroprotective effects. It has been reported that Exendin-4 upregulated FoxO 1 expression both *in vitro* and *in vivo* (Wang et al., 2017), or regulated the phosphorylation of FoxO 1 in cardiomyocytes (Eid et al., 2021). So our results about the important roles of DAF-16 in mediating the function of exendin-4 are consistent with previous studies. We can draw a conclusion from the present data that Exendin-4 has a positive effect on the FoxO family.

## Conclusion

Although Exendin-4 was reported that possesses neuroprotective effects in various AD models, we reported for the first time that Exendin-4 exhibits the ability to delay the development of paralysis and prolong the life span in the *C. elegans* model of AD. In our research, we elucidated some neuroprotective mechanisms of Exendin-4 on oxidative stress and Aβ (1-42) toxicity. The DAF-16 is required for Exendin-4 to display the antioxidant effects and neuroprotective effects. Taken together, our study testified that Exendin-4 as a marketed diabetes drug has the potential to be a kind of drug for AD.

## Data availability statement

The original contributions presented in the study are included in the article/Supplementary material, further inquiries can be directed to the corresponding authors.

## Author contributions

XS and XW designed this study. YSun, ZW, and YSu performed the experiments. YSu and ZW collected and analyzed the data. XS wrote the manuscript. XW contributed to the manuscript review. All authors contributed to the article and approved the submitted version.

## Funding

This study was supported by the Scientific and Technological Developing Scheme of Jilin Province (20190304051YY) and the Scientific Research Project of Jilin Provincial Department of Education (JJKH20210879KJ) and the Natural Science Foundation of Changchun Normal University 2018 (012).

## Conflict of interest

The authors declare that the research was conducted in the absence of any commercial or financial relationships that could be construed as a potential conflict of interest.

## Publisher's note

All claims expressed in this article are solely those of the authors and do not necessarily represent those of their affiliated organizations, or those of the publisher, the editors and the reviewers. Any product that may be evaluated in this article, or claim that may be made by its manufacturer, is not guaranteed or endorsed by the publisher.
