# Supplementary material for: Exendin-4 alleviates β-Amyloid peptide toxicity via DAF-16 in a Caenorhabditis elegans model of Alzheimer's disease
Source: Front Aging Neurosci. 2022 Aug 5;14:955113. doi: 10.3389/fnagi.2022.955113 (PMC9389237; doi:10.3389/fnagi.2022.955113)
Supplement: Supplementary file 1 [file Data_Sheet_1.docx]

Supplementary Material

**Supplementary Table 1** Paralysis statistics in CL4176 with Exendin-4 administrated

| **sample** | **mean paralysis time ±SEM(h)** | **increased paralysis%**  **(vs control)** | **Median Paralysis**  **time (h)** | **p-Value**  **(vs control)** | **p-Value summary** |
| --- | --- | --- | --- | --- | --- |
| Negative control | 44.84±0.3663 | / | 44 | / | / |
| Positive control | 47.81±0.3336 | 6.62 | 48 | P< 0.0001 | *** |
| 0.02mg/ml | 45.29±0.5613 | 1.00 | 44 | P=0.2533 | ns |
| 0.1mg/ml | 46.04±0.3964 | 2.67 | 46 | P=0.0519 | ns |
| 0.3mg/ml | 46.22±0.4381 | 3.07 | 46 | P=0.0157 | * |
| 0.5mg/ml | 47.89±0.4118 | 6.80 | 48 | P<0.0001 | *** |
| 1.2 mg/ml | 48.51±0.3666 | 8.18 | 50 | P<0.0001 | *** |

**Supplementary Table 2** Paralysis statistics in CL2006 with Exendin-4 administrated

| **sample** | **mean paralysis time ±SEM(h)** | **increased paralysis%**  **(vs control)** | **Median Paralysis**  **time (h)** | **p-Value**  **(vs control)** | **p-Value summary** |
| --- | --- | --- | --- | --- | --- |
| Negative control | 44.63±0.3618 | / | 44 | / | / |
| Positive control | 48.03±0.3319 | 7.61 | 48 | P< 0.0001 | *** |
| 0.02mg/ml | 44.80±0.4651 | 0.04 | 44 | P=0.7248 | ns |
| 0.1mg/ml | 46.31±0.4161 | 3.76 | 46 | P= 0.0059 | ** |
| 0.3mg/ml | 45.68±0.4552 | 2.35 | 46 | P= 0.0350 | * |
| 0.5mg/ml | 47.38±0.4130 | 6.16 | 48 | P<0.0001 | *** |
| 1.2 mg/ml | 48.21±0.3730 | 8.02 | 48 | P<0.0001 | *** |

**Supplementary Table 3** Lifespan statistics in CL4176 after Exendin-4 administrated

| **Sample** | **mean LS±SEM(d)** | **increased lifespan%**  **(vs control)** | **Median lifespan**  **time (d)** | **p-Value** | **p-Value summary** |
| --- | --- | --- | --- | --- | --- |
| Control | 9.42105±0.53155 | / | 8 | / | / |
| 0.02mg/ml | 10.61667±0.51645 | 12.63 | 11 | P=0.1226 | ns |
| 0.1mg/ml | 11.77966±0.5981 | 24.94 | 12 | P=0.0016 | ** |
| 0.3mg/ml | 12.44898±0.63558 | 32.05 | 13 | P=0.0002 | *** |
| 0.5mg/ml | 12.66667±0.60281 | 34.39 | 13 | P=0.0003 | *** |

**Supplementary Table 4** P value in bacteria growth curve assay

| **Exendin-4** | ***Ecoil* OP50** | | ***Ecoil* R13H8.1** | |
| --- | --- | --- | --- | --- |
|  | **p-Value** | **P Value summary** | **p-Value** | **P Value summary** |
| Control |  | / | / | / |
| 0.1mg/ml | 0.943 | ns | 0.8986 | ns |
| 0.3mg/ml | 0.8717 | ns | 0.8576 | ns |
| 0.5mg/ml | 0.8899 | ns | 0.9048 | ns |


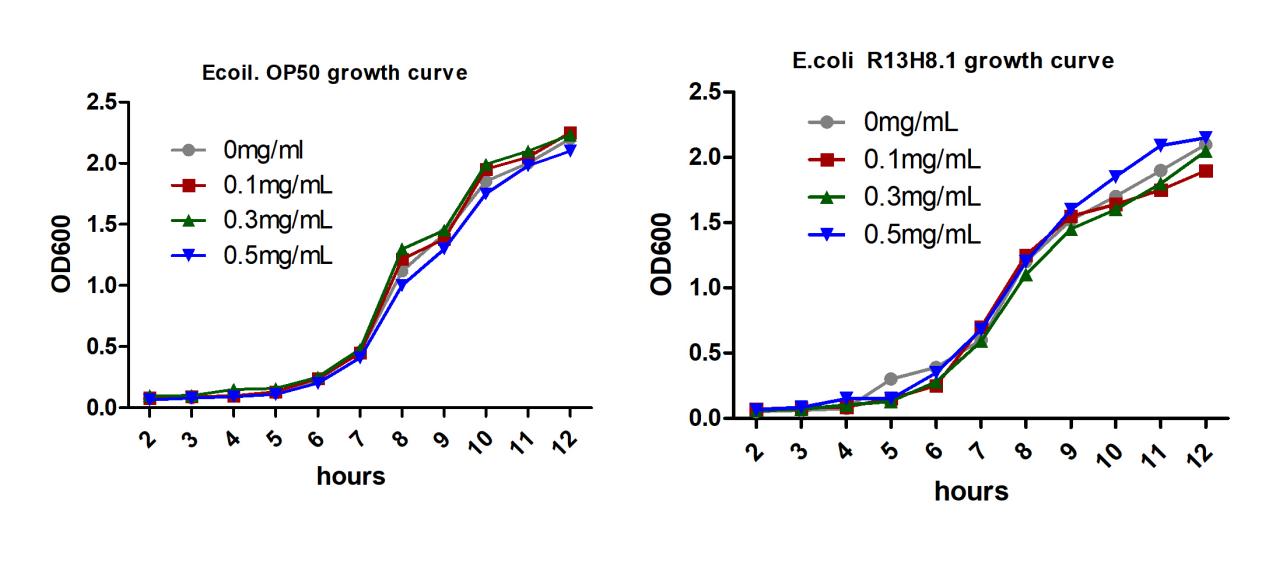


**Supplementary Figure 1.** The growth curve of *E.coil* OP50 and *E.coil* R13H8.1. Bacterial was cultured for 12 hours with the different concentrations of Exendin-4 (0.1mg/mL, 0.3mg/mL and 0.5mg/ml). Bacterial growth was monitored by detecting the optical density at 600 nm (OD600). no toxicity on the growth of *E.coli* OP50 and R13H8.1 was found.

**
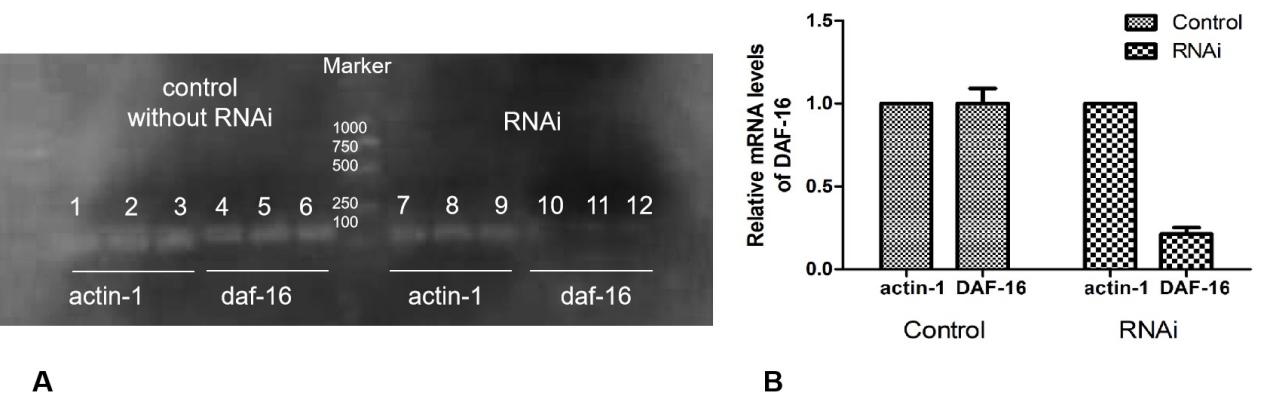
**

**Supplementary Figure 2.** DAF-16 gene RNAi efficiency assay in CL4176. (A) Agarose gel electrophoresis assay. Lane 1-3: actin-1 as internal reference without DAF-16 RNAi, Lane7-9 :actin-1 as internal reference iwith DAF-16 RNAi, Lane 4-5: DAF-16 without RNAi,Lane 10-12: DAF-16 with RNAi. (B) The mRNA levels of DAF-16 was analyzed by real time PCR. mRNA levels of DAF-16 was reduced by 29.67% after daf-16 gene knocked out.
